# Adenosine Receptor Agonist HE-NECA Enhances Antithrombotic Activities of Cangrelor and Prasugrel in vivo by Decreasing of Fibrinogen Density in Thrombus

**DOI:** 10.3390/ijms22063074

**Published:** 2021-03-17

**Authors:** Dawid Polak, Marcin Talar, Nina Wolska, Dagmara W. Wojkowska, Kamil Karolczak, Karol Kramkowski, Tomasz A. Bonda, Cezary Watala, Tomasz Przygodzki

**Affiliations:** 1 Department of Haemostasis and Haemostatic Disorders, Chair of Biomedical Sciences, Medical University of Lodz, Mazowiecka 6/8, 92-235 Lodz, Poland; dawidpolak1991@gmail.com (D.P.); marcin.talar@umed.lodz.pl (M.T.); n.m.wolska@gmail.com (N.W.); dagmara.wojkowska@umed.lodz.pl (D.W.W.); kamil.karolczak@umed.lodz.pl (K.K.); cezary.watala@umed.lodz.pl (C.W.); 2 Department of Physical Chemistry, Medical University of Bialystok, Mickiewicza 2A, 15-089 Bialystok, Poland; kkramk@wp.pl; 3 Department of General and Experimental Pathology, Medical University of Bialystok, Mickiewicza 2C, 15-222 Bialystok, Poland; tomasz.bonda@umb.edu.pl

**Keywords:** adenosine receptors, P2Y_12_ inhibitors, platelets, thrombosis, animal models

## Abstract

Blood platelets’ adenosine receptors (AR) are considered to be a new target for the anti-platelet therapy. This idea is based on in vitro studies which show that signaling mediated by these receptors leads to a decreased platelet response to activating stimuli. In vivo evidence for the antithrombotic activity of AR agonists published to date were limited, however, to the usage of relatively high doses given in bolus. The present study was aimed at verifying if these substances used in lower doses in combination with inhibitors of P2Y_12_ could serve as components of dual anti-platelet therapy. We have found that a selective A_2A_ agonist 2-hexynyl-5’-N-ethylcarboxamidoadenosine (HE-NECA) improved the anti-thrombotic properties of either cangrelor or prasugrel in the model of ferric chloride-induced experimental thrombosis in mice. Importantly, HE-NECA was effective not only when applied in bolus as other AR agonists in the up-to-date published studies, but also when given chronically. In vitro thrombus formation under flow conditions revealed that HE-NECA enhanced the ability of P2Y_12_ inhibitors to decrease fibrinogen content in thrombi, possibly resulting in their lower stability. Adenosine receptor agonists possess a certain hypotensive effect and an ability to increase the blood–brain barrier permeability. Therefore, the effects of anti-thrombotic doses of HE-NECA on blood pressure and the blood–brain barrier permeability in mice were tested. HE-NECA applied in bolus caused a significant hypotension in mice, but the effect was much lower when the substance was given in doses corresponding to that obtained by chronic administration. At the same time, no significant effect of HE-NECA was observed on the blood–brain barrier. We conclude that chronic administration of the A_2A_ agonist can be considered a potential component of a dual antithrombotic therapy. However, due to the hypotensive effect of the substances, dosage and administration must be elaborated to minimize the side-effects. The total number of animals used in the experiments was 146.

## 1. Introduction

Antiplatelet therapies decrease the ability of blood platelets to form occlusive thrombus and hence limit the occurrence of such pathological conditions as stroke or heart attacks. Antiplatelet drugs act through molecular targets such as purinergic receptors P2Y_12_, integrin α_IIb_β_3_, cyclooxygenase-1 or protease activated receptors. In selected indications, dual therapy is applied where cyclooxygenase-1 inhibition is accompanied by a P2Y_12_ inhibitor. The effectiveness of such dual strategy stems from the fact that two distinct molecular pathways which lead to platelet activation are compromised. Based on this concept, new dual antiplatelet strategies can be conceived.

A target, which has recently been considered in the context of dual therapy, is adenosine receptors (AR). They are expressed on blood platelets as two sub-types: A_2A_ and A_2B_ [1]. Their naturally occurring ligand is adenosine, a purine metabolite that is present in plasma mainly due to the activity of ecto-5′-nucleotidaze (CD73), which hydrolyses adenosine monophosphate. Activation of these G-coupled receptors leads to increased adenylyl cyclase activity with a concomitant increase in intracellular cAMP and, consequently, to the inhibition of platelet activation and aggregation [2,3]. As such, adenosine receptor agonists possess an antiplatelet effect, as proven in vitro, and have been found to act as antiplatelet [4] and antithrombotic agents in vivo [5,6,7] when applied in high doses of 29 up to 200 mg/kg b.w. [5,6,7,8]. Their use in such high doses is potentially compromised by their systemic effects such as hypotension or increasing of the blood–brain barrier permeability. However, if we consider these substances as components of dual antiplatelet therapy, their effective doses could be lowered, which could result in a limitation of their side-effects. Their potential to be a component of dual antiplatelet therapy was shown by our group in an in vitro model in combination with P2Y_12_ inhibitors [9,10]. The present study is aimed at providing evidence for the in vivo effectiveness of such a dual approach.

As an A_2A_ selective agonist, the present study uses HE-NECA, a derivative of the non-selective AR agonist NECA. HE-NECA has proven antiplatelet activity in vitro [11]. As representative compounds of P2Y_12_ inhibitors, cangrelor and prasugrel have been chosen. These substances differ in the routes of their administration and their metabolic fates in an organism. Cangrelor blocks ADP signaling in a direct, reversible, and competitive way [12] and it is administered intravenously during percutaneous coronary intervention. Prasugrel, in turn, is a prodrug which is administered orally in a chronic manner. This irreversible antiplatelet agent is rapidly converted to its active metabolite by the P450 cytochrome [13,14]. The in vivo anti-thrombotic activities of the tested substances were assayed with the use of a mouse model of chemically induced thrombosis.

To better characterize the mode of action of AR agonists on platelet aggregation during primary hemostasis, a model of in vitro thrombus formation under flow conditions was used.

Finally, the effects of the tested doses of HE-NECA on the blood–brain barrier permeability and hypotension were assessed, as these physiological effects occur as a consequence of the systemic application of AR agonists.

## 2. Results

### 2.1. Combined Effect of HE-NECA and P2Y_12_ Inhibitors on Platelet Aggregation in Whole Blood

The anti-aggregatory effect of adenosine receptor agonist HE-NECA was tested using human whole blood stimulated with 10 μM ADP. A dose-response, non-linear regression curve was plotted, yielding a curve with the maximal inhibition value of 84.7 ± 4.9%, and IC_50_ of 0.2 µM (95% confidence interval: 0.06 to 0.37) with a coefficient of determination (R^2^) equal to 0.780.

HE-NECA was used in a combination with two P2Y_12_ receptor antagonists: either cangrelor or prasugrel metabolite R-138727 (PM). Each compound was used in its IC_50_ (previously published values: cangrelor 17 nM, and PM 1.3 µM [9]).

**Figure 1:**
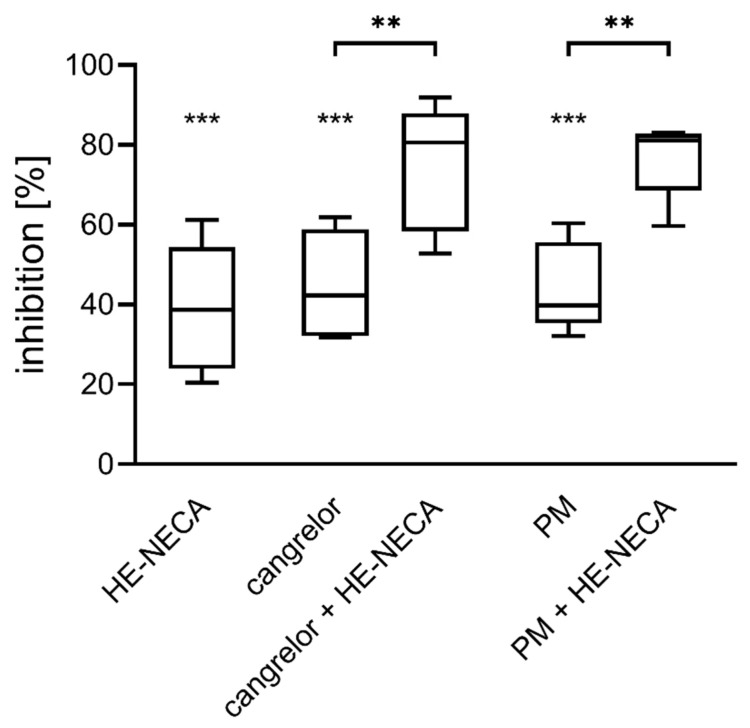
HE-NECA, the adenosine receptor agonist, intensifies the anti-aggregatory effect of P2Y_12_ antagonists. Data are presented as median, interquartile range and minimum and maximum values (*n* = 5). Changes in platelet aggregation were measured in whole blood in response to 10 μM ADP after 3 min preincubation at 37 °C with HE-NECA and/or cangrelor, or 15 min preincubation at 37 °C with PM (prasugrel metabolite). Statistical significance was estimated by two-way ANOVA with Sidak’s multiple comparisons *post-hoc* test (only relevant comparisons were tested). ** *p* < 0.01, *** *p* < 0.005.

HE-NECA and P2Y_12_ antagonists significantly reduced platelet aggregation (Figure 1). Simultaneous application of an adenosine receptor agonist was found to intensify the inhibitory effect of P2Y_12_ on platelet aggregation. A 38% median increase in the inhibition rate was observed for cangrelor and 41% for PM, when the substances were combined with HE-NECA compared to their activities alone.

### 2.2. Ferric Chloride-Induced Thrombosis—Microscopy

**Figure 2:**
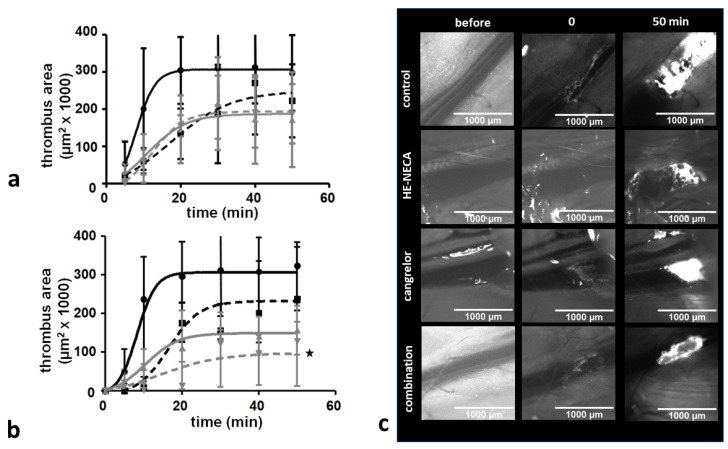
Effects of cangrelor and HE-NECA applied alone and in a combination on the size of thrombus induced with the use of FeCl_3_ in the jugular vein in mice. (**a**) Circles—DMSO, squares—HE-NECA 2 mg/kg b.w., triangles—cangrelor 0.1 mg/kg b.w., inverted triangles—cangrelor + HE-NECA. (**b**) Circles—DMSO, squares—HE-NECA 4 mg/kg b.w., triangles—cangrelor 0.2 mg/kg b.w., inverted triangles—cangrelor + HE-NECA. Data are shown as median with IQR, *n* = 5–7. Curves were fitted with the four-parametric equation with a shared bottom value, using transformed data. Kruskal–Wallis test followed by Dunn’s multiple comparison test were used to compare thrombi area 50 min. after initialization of thrombus formation. * *p* < 0.05 (corrected for multiple comparisons). Panel (**c**) shows exemplary images of thrombi formed in mice treated with DMSO or with a combination of HE-NECA 4 mg/kg b.w. and cangrelor 0.2 mg/kg b.w.

The antithrombotic effects of HE-NECA in combination with cangrelor on FeCl_3_-induced thrombosis were determined using intravital microscopy. As shown in Figure 2 when HE-NECA and cangrelor were applied in doses of 2 mg/kg b.w. and 0.1 mg/kg b.w. respectively, their effect on thrombus area was not significant. Thrombus growth was significantly inhibited by the combination of the substances when used in doses of 4 mg/kg b.w. and 0.2 mg/kg b.w., respectively. Neither of the substances demonstrated a significant effect when used alone. However, microscopic observations found that in many cases, the thrombus did not stop the blood flow entirely despite seeming to occupy the whole lumen of the vessel. The thrombi appeared to differ regarding their level of compactness and permeability, and such features are difficult to quantitatively assess based on wide-field microscopy images. Therefore, assessment of thrombus size did not provide relevant information on its occlusive properties. The latter could be more relevantly evaluated by assessing blood flow in the injured vessel. This approach was applied in further studies with the use of laser Doppler flowmetry.

The number of mice used in this part of experiment was 36.

### 2.3. Ferric Chloride-Induced Thrombosis—Laser Doppler Flowmetry

**Figure 3:**
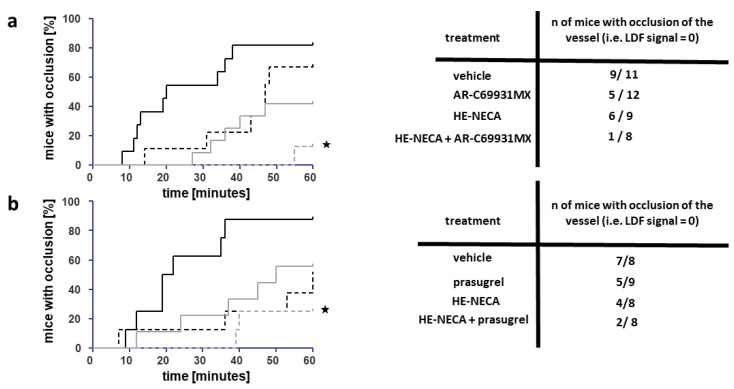
Effects of cangrelor and HE-NECA (**a**) or prasugrel and HE-NECA (**b**) applied alone or in a combination on occlusion induced with the use of FeCl_3_ in the jugular vein in mice. Left panels show cumulative curves of the percentage of mice in which occlusion occurred in the function of time, solid black line: DMSO, black dashed line: HE-NECA, gray solid line: cangrelor (**a**) or prasugrel (**b**), gray dashed line: combination of HE-NECA and cangrelor (**a**) or prasugrel (**b**); *n* = 8–11. The significance of differences between curves was estimated with the use of log-rank test with the correction for multiple comparisons, * *p* < 0.05 (corrected). Right panels present ratios of animals in which total occlusion occurred. Significance of differences between the ratios were tested with the use of an exact Fisher *t*-test. The exact *p*-values are as follows: (**a**) vehicle vs. cangrelor *p* = 0.06024; vehicle vs. HE-NECA *p* = 0.39551; vehicle vs. a combination *p* = 0.00488; HE-NECA vs. a combination *p* = 0,03640; cangrelor vs. a combination *p* = 0,18731 and (**b**) vehicle vs. prasugrel *p* = 0.18326; vehicle vs. HE-NECA *p* = 0.14103; vehicle vs. combination *p* = 0.02028; HE-NECA vs. a combination *p* = 0.30420; prasugrel vs. a combination *p* = 0.21781.

Bolus application of a combination of HE-NECA and cangrelor in doses which turned out to be effective in the above-described experiment resulted in a prolongation of a time to occlusion when compared to the animals treated with a vehicle; however, the effect was not significant when the substances were applied alone (Figure 3a). Although treatment with the combination of pharmaceuticals did not result in any significant prolongation of the time to occlusion compared to the use of individual agents, such differences were detected when bootstrap methods were used; this might suggest that the observed lack of difference was due to the low sample size. A lower ratio of total occlusion was observed in the combination group than the vehicle group (Figure 3a). The ratio did not significantly differ from the vehicle-treated animals when the substances were applied alone.

Chronic application of HE-NECA and prasugrel resulted in prolongation of the time to occlusion compared to the control when applied in combination, but not when applied individually (Figure 3b). Similarly to the results of bolus application of cangrelor and HE-NECA, the time to occlusion in animals treated with the combination was not significantly prolonged when compared to animals treated with the individual substances. Again, such differences were detected when bootstrap methods were used, which suggests that the lack of difference was most likely due to low sample size. The group which received a combination of drugs did not demonstrate a significantly lower ratio of total occlusion than the group treated with a vehicle, when *p*-value was corrected for multiple comparisons.

The number of mice used in this part of the experiment was 73.

### 2.4. Platelet Activation and Thrombus Formation In Vitro

**Figure 4:**
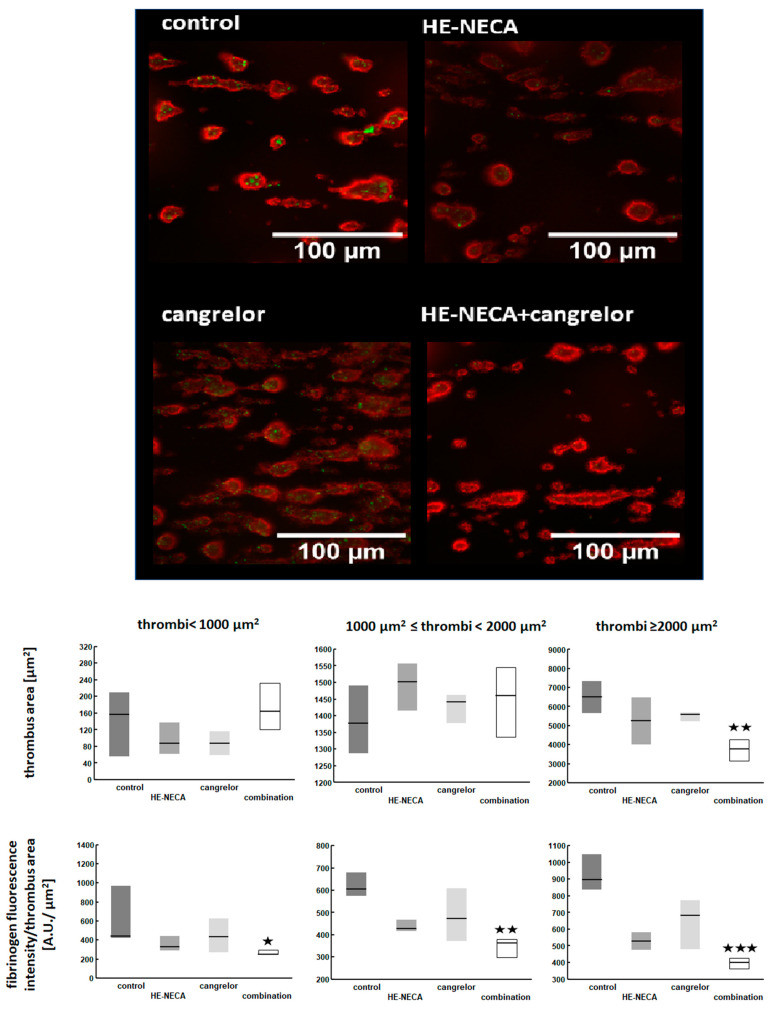
Effects of cangrelor and HE-NECA applied alone or in a combination on thrombi formed in vitro under flow conditions. Representative images show thrombi (red) formed under flow conditions and fibrinogen deposits (green). Area of individual thrombi and fibrinogen deposition in these thrombi presented as median with IQR, *n* = 6, statistical significance was tested with Friedman’s test followed by the Dunn’s multiple comparison test (only relevant comparisons were tested). * *p* < 0.05 (corrected), ** *p* < 0.01 (corrected), *** *p* < 0.001 (corrected).

Area of individual thrombi larger than 2000 µm^2^ formed under flow conditions were decreased by a combination of HE-NECA and cangrelor when compared to control, while the drugs given alone did not have a significant effect (Figure 4). The size of smaller thrombi was not affected neither by the drugs alone or when used in combination. Fibrinogen density in individual thrombi was lower in the presence of a combination of HE-NECA and cangrelor than in control in an entire range of thrombi sizes (Figure 4).

### 2.5. The Blood–Brain Barrier Permeability

**Figure 5:**
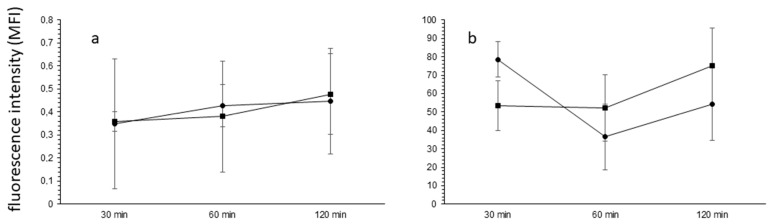
Effects of HE-NECA on the in vivo blood–brain barrier permeability measured by the assessment of fluorescent dye concentration in brain tissue after peripheral dye administration. The fluorescent dyes were administered by an intraperitoneal injection and their concentrations detected in brain tissue homogenates in a time course. (**a**) Evans blue (*n*= 4) or (**b**) fluorescein (*n*= 5) fluorescence in brain tissue homogenates from mice treated with HE-NECA in a dose of 4 mg/kg b.w. Circles: vehicle-treated animals, squares: HE-NECA-treated animals. Results are presented as mean and standard deviation. Two-way ANOVA followed by Tukey’s *post-hoc* test did not detect statistical significance of differences.

The potential effect of HE-NECA on the blood–brain barrier integrity was investigated in vivo in a mouse model. As shown in Figure 5, HE-NECA administration at a dose of 4 mg/ kg b.w. did not change the functional state of the blood–brain barrier (BBB). No statistically significant differences in the concentration of both tested fluorescent dyes were observed in animal brain homogenates after HE-NECA administration.

The number of mice used in this part of experiment was 18.

### 2.6. Blood Pressure Assessment

**Figure 6:**
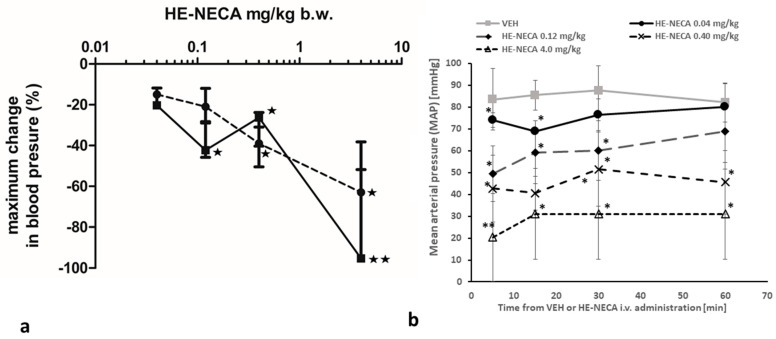
(**a**) Effects of HE-NECA on systolic (SBP) and diastolic (DBP) blood pressure in mice. Data, shown as median with IQR (*n* = 4–5), present the maximum change in diastolic (circles) and systolic (squares) blood pressure in comparison to the initial blood pressure (measured prior to HE-NECA injection). A Mann–Whitney U test was used to evaluate statistical significance: * *p* < 0.05, ** *p* < 0.01. For a vehicle-treated group (concentration of HE-NECA equal to 0) ΔSBP and ΔDBP were 8.55 ± 4.11 and 12.50 ± 0.96 mmHg, respectively. (**b**) Mean Arterial Pressure (MAP) changes within 60 min after HE- NECA i.v. treatment in ketamine/xylazine anesthetized mice. Note that VEH values are diminished due to anaesthesia. Data are shown as a Mean ± SD; * *p* < 0.05; ** *p* < 0.01 vs VEH; *n* = 4–5.

The effect of HE-NECA on blood pressure was assessed using a non-invasive tail-cuff method in the anesthetized mice. As shown in Figure 6, HE-NECA significantly decreased systolic blood pressure (SBP) in a dose-dependent manner at doses of 4, 0.4, and 0.12 mg/kg b.w., and diastolic blood pressure (DBP) at doses of 4 and 0.4 mg/kg b.w., when compared to mice injected with a vehicle.

Furthermore, the duration of the hypotensive activity was also found to be dose-dependent: the effects lasted longer than one hour at doses of 4 and 0.4 mg/kg b.w, between 15 min and one hour at 0.12 mg/kg b.w. and up to fifteen minutes at 0.04 mg/kg b.w and 0.01 mg/kg b.w.

The number of mice used in this part of the experiment was 19.

## 3. Discussion

### 3.1. The Antithrombotic Activity of HE-NECA in Combination with Cangrelor or Prasugrel

The presented work shows that AR agonists significantly enhance an effect of commonly used antiplatelet drugs in vivo. These studies are a continuation of our previously published results, which showed such an enhancement in vitro [9,10,15].

Although the presented studies are not the first to report antithrombotic effects of AR agonists in vivo, they provide a significant new input to the previously published reports. First of all, previous in vivo studies dealt with the antithrombotic effects of isolated AR agonists. Another crucial difference relates to the doses used: our studies involved lower doses of AR agonists than the doses used up to date, i.e., 4 mg/kg b.w. vs 29–200 mg/kg b.w. [5,6,7,8] Finally, an assessment of thrombus formation in our studies was not limited to the evaluation of the thrombus size by intravital microscopy, but also utilized the functional assessment of the occlusive properties of thrombus. It should be underlined that methods based on FeCl_3_ application engage mechanisms of platelet activation regardless of arterial or venous location [16]. Therefore, in the presented experiments blood platelets are the main factor responsible for thrombus formation.

The present in vivo study uses HE-NECA as a representative compound of selective A_2A_ agonists; it was chosen based on the results of in vitro studies, which found the combination of HE-NECA with cangrelor or prasugrel metabolite to have over-additive effects. The choice of P2Y_12_ inhibitors was due to the differences in the way of administration and metabolism. Our idea was to test the efficacy of the model of AR agonist with two distinct members of the family of P2Y_12_ inhibitors. The regimen of administration of the P2Y_12_ inhibitors in vivo was chosen to mimic those used in clinical applications: cangrelor was applied by a bolus intravenous injection, while prasugrel was administered chronically. A significant prolongation of the time to occlusion was observed for the combination of both of P2Y_12_ inhibitors with HE-NECA confirming that platelet A_2A_ adenosine receptors can be considered auxiliary targets in antiplatelet therapy.

The mechanism of anti-platelet action of AR agonists has been to a large extent explained by previous in vitro studies. The substances reduce intraplatelet calcium mobilization, α_IIb_β_3_ activation and exogenous fibrinogen binding. Our previous in vitro studies have shown that by doing so they deepen the effects of P2Y_12_ inhibitors [15]. In the presented studies we further confirm these findings in functional assays by showing that thrombi formed in the presence of HE-NECA and cangrelor contained less fibrinogen than thrombi formed in the presence of isolated substances. This resulted in a decrease of the size of the largest thrombi, while the size of thrombi smaller than 2000 µm^2^ was unaffected by the combination of the substances. In turn, the density of fibrinogen was decreased by HE-NECA in combination with cangrelor in the entire range of the size of thrombi. Therefore it may be deduced that while the smaller thrombi were still resistant to the shear forces despite decreased fibrinogen content, the larger structures could no longer build-up in these conditions.

Since the in vitro experiments were performed in the presence of thrombin inhibitor, the fibrinogen present in thrombi was more likely a platelet-bound fraction than fibrin deposition. Thus, instability of large thrombi was presumably due to a decreased strength of interplatelet interactions, which depend on integrin α_IIb_β_3_-bound fibrinogen.

The finding that AR agonists modulate the activity of P2Y_12_ inhibitors sheds new light on the interaction between these antiplatelet drugs and naturally occurring antagonists of A_2A_ receptors, such as caffeine, theophylline or theobromine. It has been shown that chronic caffeine intake by humans increases the platelet sensitivity of A_2A_ receptors towards HE-NECA [17,18]. Consequently, caffeine intake was found to enhance the anti-platelet effect of clopidogrel in patients with coronary artery disease [19]. Although the exact mechanism by which the antagonists of A_2A_ sensitize platelets towards agonists of these receptors is not known, it is suggested that the inhibition of these receptors might lead to their increased expression in megakaryocytes [17,18]. Platelets which stem from these megakaryocytes may, as a consequence, present higher sensitivity towards the endogenous A_2A_ agonist adenosine. It was suggested that caffeine intake may therefore contribute to inter- and intra-patient variability in the response to P2Y_12_ inhibitors [19]. Our finding that A_2A_ receptor activation in fact modulates the antithrombotic effects of P2Y_12_ inhibitors suggests that the sensitivity of platelet A_2A_ receptors may influence patient response to these antiplatelet drugs.

Interestingly, agonists of A_2A_ receptors can prevent thrombus formation in vivo not only by activating the platelet pool of the receptor. It has been shown that activation of these receptors on polymorphonuclear neutrophils (PMNs) decreases their adhesion to the site of vascular injury [20]. Since PMNs take part in a production of fibrin during thrombus formation [21], a decrease in their accumulation in the site of injury evidently contributes to less effective thrombosis. This process has also been shown to be important in venous thrombosis [22]. The pleiotropic activity demonstrated by A_2A_ receptors agonists in thrombosis further indicates their potential as antithrombotic agents.

The presented studies have a certain limitation: we have not performed the bleeding time assays which evaluate overall blood clotting capacity in conditions of blood extravasation. This test is usually used to assess the undesirable effects of a tested treatment on blood clotting ability in conditions where the blood vessel wall integrity is disrupted. An ideal antiplatelet drug should spare this basic hemostatic function. The reason why we omitted these tests is that the presented studies are proof of concept experiments rather than evidence of the superiority of HE-NECA over other anti-platelet drugs in terms of the limitation of side-effects. We do not expect that HE-NECA per se could become a drug. The compound was used as a potent representative of A2A agonists. Bleeding tests will be performed in the future with A2A agonists characterized by lower hypotensive effects, which are currently under investigation.

### 3.2. HE-NECA Effect on Blood Pressure

One potential drawback of using of A_2A_ agonists as antiplatelet agents is their considerable hypotensive effect. A dose of HE-NECA given in bolus, which demonstrated an anti-thrombotic activity, also had a strong hypotensive effect, as indicated by the measurements of blood pressure. A dose of 4 mg/kg caused pronounced hypotension, reaching the lower detection limits of the method (SBP/DBP < 40/20 mmHg). Despite this drop in blood pressure, which could indicate cardio-vascular collapse, both laser Doppler flowmetry and intravital microscopy confirmed a continued blood flow and the formation of thrombus. Such a strong hypotensive effect, however, is undoubtedly an important limitation of the experimental setup. It is important to emphasize that the dose of AR agonist used in our studies (4 mg/kg b.w.) was much lower than those used in previous studies on anti-thrombotic activity of AR agonists; in addition, unlike the present study, these studies did not clearly report the effects of the tested compounds on blood pressure.

The hypotensive effect could be obviously limited by decreasing the dose and extending the administration period of the substance. Our results demonstrate that chronic administration of HE-NECA effectively limited thrombus formation. Assuming that the entire substance was absorbed from the peritoneum, the application of 8 mg/kg b.w. /day would result in a dose of approximately 0.3 mg/kg b.w./hour. This dose, when applied in bolus, resulted in an approximate 30% decrease of blood pressure, which was shorter than one hour. Therefore, the administration of lower doses, applied chronically, may minimize the adverse effects and retain the anti-thrombotic activity. This finding has an important implication for studies of the effects of AR agonists on thrombus formation.

### 3.3. HE-NECA Effect on the Blood–Brain Barrier Permeability

Another side-effect of A_2A_ agonists, which can hinder their application as anti-thrombotic drugs, is that they increase the blood–brain barrier permeability. It has been shown that the specific A_2A_ agonist regadenoson and non-specific adenosine receptor agonist NECA increased the permeability of an endothelial cell monolayer in vitro [23]. In vivo studies in rats have shown that NECA increased the blood–brain barrier permeability [24] and improved the brain permeability for anticonvulsive drugs [25]. Importantly, the effect of NECA on blood–brain barrier permeability was not linearly dependent on a dose: its effectiveness decreased at doses higher than 0.3 mg/kg b.w. This was explained by the receptor desensitization by such higher doses [24]. Similarly, regadenoson increased the blood–brain barrier permeability in rats for temozolomide, a chemotherapeutic drug used in neuro-oncology [26]. In contrast, CGS21680—an A_2A_ agonist—decreased BBB permeability in a model of experimental autoimmune encephalomyelitis in mice [27]. Human studies using regadenoson at doses approved for pharmacologic cardiac stress testing did not yield any significant effect on blood–brain barrier permeability for contrast agents [28] or temozolomide [29]. In our present study, HE-NECA did not significantly increase the blood–brain barrier permeability, when applied at a dose which had an antithrombotic effect. This might be, however, the effect of the receptor desensitization described above.

## 4. Conclusions

Currently used P2Y_12_ inhibitors present relatively high efficacy. A fraction of patients who do not respond to the therapy with ticagrelor and prasugrel is lower than in case of patients treated with clopidogrel [30,31]. And yet still a group of patients responds poorly to these drugs. Our studies along with other reports published previously suggest that platelet A_2A_ receptors can be considered as targets of supplementary therapy. However, due to the hypotensive effect of the substances, dosages and administration must be elaborated to minimize the side-effects.

## 5. Materials and Methods

### 5.1. Chemicals

The adenosine receptor agonist—HE-NECA (CAS № 141018-30-6) was purchased form Abcam (Cambridge, United Kingdom), and cangrelor (AR-C69931MX) from Cayman Chemical (Ann Arbor, MI, USA). Prasugrel and prasugrel metabolite (R-138727) were purchased from BOC Sciences (Shirley, NY, USA). Dimethyl sulfoxide (DMSO) and adenosine diphosphate (ADP) were obtained from Sigma (St. Louis, MO, USA). Ferric chloride (FeCl_3_) was obtained from POCh (Gliwice, Poland). Oregon Green 488-labelled human fibrinogen was from Molecular Probes (Eugene, OR, USA). Type I collagen from equine tendon was purchased from Chrono-log Corp. (Havertown, PA, USA) D-phenylalanyl-prolyl-arginyl chloromethyl ketone (PPACK), was from Calbiochem (Darmstadt, Germany). Anti-CD41 phycoerythrin (PE)-conjugated antibodies were purchased from Becton-Dickinson (San Diego, CA, USA).

A 100 mM stock solution of HE-NECA was prepared in DMSO. During platelet aggregometry measurements, aliquots of the stock solution were then diluted with PBS to working concentrations not exhibiting precipitates, as noted by Boncler et al. [9], and added to the biological material. The dilution factor was chosen in such a way to yield a maximal concentration of DMSO never exceeding 0.1% in a biological sample in any of the assays.

### 5.2. Animals

C57BL/6 male and female mice aged 8–12 weeks were used for studies. Animals were obtained from the University of Lodz, Poland. The studies were approved by the Local Ethical Committee on Animal Experiments, Medical University of Lodz (approval numbers: 63/ŁB119/2018, approval date 3 December 2018 and 20/ŁB133/2019, approval date 20 May 2019). Animals had access to food and fresh water ad libitum. All efforts were made to minimize animal suffering.

Minimal sample sizes in animal studies were estimated as follows. Comparison of the kinetics of thrombus growth based on intravital microscopy: to detect the standardized effect of 2.5 (a measure of the effects in each method is based on our previous experiments) with *p* < 0.013 (corrected for multiple comparisons) with the power of 0.8, the minimum sample size is *n* = 5.

In the assessment of the fraction of animals in which an occlusion occurred to detect the standardized effect of 2 with *p* < 0.013 (corrected for multiple comparisons) with the power of 0.8, the minimum sample size is *n* = 7.

In the assessment of the blood–brain barrier permeability and blood pressure to detect the standardized effect of 2 with *p* < 0,05 with the power of 0.8, the minimum sample size is *n* = 4.

### 5.3. Blood Donors

After having received written consent from volunteers, blood was collected from healthy donors (*n* = 5; four women, one man; mean age 32.8 ± 8.4 years), who stated that they had not taken medications known to influence platelet function for at least two weeks prior to the study, into a vacuum tube containing 0.105 mol/l buffered sodium citrate, with a final citrate: blood ratio of 1:9 vol/vol. Experiments were approved by the Ethics of Research in Human Experimentation Committee at the Medical University of Lodz, approval number (RNN/43/17/KE approval date 14 February 2017).

### 5.4. Platelet Aggregometry Measured in Whole Blood

Measurements of platelet aggregation were performed in a Multiplate analyzer (Roche Holding AG, Hoffmann-La Roche, Basel, Switzerland) according to the manufacturer’s instruction. Whole blood was preincubated with HE-NECA and/or P2Y_12_ inhibitor (cangrelor or prasugrel metabolite, PM) for 3 (HE-NECA and cangrelor) or 15 (prasugrel metabolite) minutes at 37 °C; then 300 µL aliquot of blood was transferred into a measurement cell, diluted with 300 μL saline (0.9%) and preheated to 37 °C for another three minutes. Then, ADP (the final concentration of 10 µM) was added and platelet aggregation was recorded continuously for 10 min. Area under the curve (AUC) was analyzed. All measurements were completed within three hours of blood collection.

### 5.5. HE-NECA and Cangrelor Administration

HE-NECA (4 mg/kg b.w.) and cangrelor (0.2 mg/kg b.w.) were administered alone or in combination by an injection into the retro-orbital sinus in 100 µL aliquot of saline. The injected volume always contained 2.5% DMSO, regardless of the combination of the composition of the tested substances. This amount of DMSO corresponds to a dose of approximately 100 µL/kg b.w., which is in the range of the tolerability of the solvent [32]. Dosing of cangrelor was chosen on the basis of preliminary experiments, with the aim of approximately 50% inhibition of occlusion. The dose used was approximately 50% lower than that used in clinical application: the currently used clinical dosage of cangrelor is 30 µg/kg b.w. followed by the infusion of 4 µg/kg b.w./min for at least two hours [33]. This results in a dose of at least 510 µg/kg b.w., while our dose was 200 µg/kg b.w.

### 5.6. HE-NECA and Prasugrel Administration

Micro-osmotic pumps (model 1007D, Alzet, Cupertino, CA, USA) were filled with solutions of HE-NECA, prasugrel or a combination thereof. Concentrations of the substances were adjusted in such a way that their release resulted in dosages of 8 mg/kg b.w./day of HE-NECA and 0.4 mg/kg b.w./day of prasugrel. This route of administration of P2Y_12_ inhibitors was previously used and proved effective in animal studies [34]. The pumps were aseptically implanted intraperitoneally through an incision in the abdomen. The dose of prasugrel was chosen on the basis of literature data showing that 0.3 mg/kg/b.w. prasugrel applied in a single oral dose resulted in a decreased, but not abolished thrombus formation in mice, while 1 mg/kg/b.w abolished thrombus formation in over 60% of the mice [35].

### 5.7. Measurement of Thrombus Formation with the Use of Intravital Microscopy

Chemical injury of the endothelium by FeCl_3_ is a well-accepted experimental model to study thrombosis [36]. FeCl_3_ causes severe damage in all layers of the vessel wall without rupturing the internal elastic lamina, and after the application of FeCl_3_, numerous spherical bodies appear to be budding off the endothelium into the vessel lumen [36]. Hence, only the basement membrane components are exposed to circulating blood cells.

Male C57BL/6 mice were anesthetized with xylazine in a dose of 20 mg/kg b.w. and ketamine in a dose of 100 mg/kg b.w i.m., injected with platelet-specific fluorescent anti-GPIbβ antibodies at a dose of 0.1 µg/g b.w. and placed on the stage of an upright microscope equipped with saline immersion objectives. The right femoral vein was revealed by skin incision. Superfusion with saline at a constant temperature of 37 °C was assured by a peristaltic pump. A vascular injury was generated by applying a filter paper saturated with 12.5% FeCl_3_ on a top of the femoral vein for 30 s. Images were taken at 0, 5, 10, 20, 30, 40, 50 min. The size of a thrombus was assessed in the images with the use of FIJI software [37].

### 5.8. Measurement of Thrombus Formation with the Use of Laser Doppler Flowmetry (LDF)

Male C57BL/6 mice were anesthetized as described above. The animal was placed on a surgical table to control the body temperature, and the right jugular vein was exposed. A vascular injury was generated by applying a filter paper saturated with 12.5% FeCl_3_ on top of the right jugular vein for the duration of the experiment. To determine the time to occlusion, a flow probe connected to a laser Doppler flowmeter (model ML191, ADInstruments, Colorado Springs, CO, USA) was fixed in a holder, allowing the positioning and mechanical stabilization, and was placed over the jugular vein distally from the site of the injury. The distance between the tip of the probe and the vessel was approximately 2 mm. Blood flow was recorded. In a fraction of animals, as an effect of FeCl_3_ exposition, Doppler arbitrary units (LDU) reached values close to 0 and fluctuated around this value for a certain period of time. To avoid an excessive period of observation, an endpoint of the experiment has been defined [38,39]. Occlusion was defined as a drop of the laser LDU value to less than 10% of an initial LDU value for a period of at least 1 min.

### 5.9. Assessment of HE-NECA and P2Y_12_ Inhibitors Effect on Thrombus Formation under Shear Stress In Vitro

Effect of HE-NECA and cangrelor on thrombi formation was assayed with the use of the Venaflux platform (Cellix, Dublin, Ireland). The channels of Vena8 Fluoro+ biochip were coated with type I collagen (20 µg/mL) overnight at 4 °C and blocked with 0.1% BSA for 1 h at 4 °C. Biochip was mounted on a stage of an inverted AxioVert microscope thermostatically controlled throughout the experiment at 37 °C (Carl Zeiss, Oberkochen, Germany). Samples were added with the tested substances, OregonGreen-conjugated fibrinogen (30 µg/mL), recalcified with CaCl_2_ (2 mM) and thrombin inhibitor PPACK (62.5 µM) was added shortly before the measurement. The samples were then perfused using a shear force of 60 dynes/cm^2^ for 2 min. The thrombi in the channels were stained by filling the channels with antiCD41 PE-conjugated antibodies. Thereafter the channels were flushed with CellFix for 2 min at 5 dynes/cm^2^. Thrombi and fibrinogen were visualized with the use of an AxioObserver microscope (Carl Zeiss, Oberkochen, Germany).

The images of thrombi were segmented and the area of individual thrombi were assessed with use of Ilastik software [40]. The maps of segmented thrombi were used to quantify fibrinogen fluorescence intensity in each individual thrombus with the use of FIJI software [37]. Normalization of fibrinogen fluorescence intensity in each individual thrombus against its area was performed with the use of scripts written in Python.

### 5.10. The Blood–Brain Barrier Permeability In Vivo

Animals received intraperitoneally an HE- NECA solution at a dose of 4 mg/kg b.w. for 30, 60 or 120 min. Additionally, 30 min before the end of the experiment, animals were also intraperitoneally injected with a fluorescent dye solution. Two dyes were used: fluorescein (a molecular weight 300 Da, at the dose 100 mg/mL in PBS), which indicates a slight disruption of BBB, and Evans blue combined with an albumin (a molecular weight 65 kDa, at the dose 10 mg/mL in PBS), which indicates a severe disruption of the blood–brain barrier. After an indicated time period, the mice were anesthetized intraperitoneally with ketamine and xylazine and perfused through the left cardiac ventricle with the ice cold PBS and the brains were collected. As a control, mice that received the HE-NECA solvent—DMSO, were used.

The collected brain tissue fragments were placed in methanol and homogenised. To 1 mL of the brain tissue homogenates from the animals injected with fluorescein, 20% TCA in H_2_O was added. Next, the samples were centrifuged for 5 min, 1250× *g*, 4 °C and incubated for 24 h in 4° C. To the brain tissue, homogenate samples from the animals injected with Evans blue, 100% TCA in H_2_O were added in a 1:1 v/v ratio. The samples were then incubated for 24 h at 37 °C. After the incubation, the fluorescence levels were measured at λ 480_ex_/538_em_ in the samples with fluorescein, and at λ 620_ex_/680_em_ in the samples with Evans blue.

### 5.11. Blood Pressure Assessment

Systolic and diastolic blood pressure (SBP/DBP) was measured in the anesthetized mice using a tail-cuff method with the ADInstruments Apparatus with LabChart 7.3.8 software, as described earlier [41]. After anesthesia (ketamine + xylazine: 120 mg/kg, i.p., Ketamina 10%, Biowet, Poland; 12.5 mg/kg, i.p., Xylapan, Biowet, Poland) the left femoral vein was isolated and the measurement of basal blood pressure was performed. Then, either a vehicle (0.9%NaCl with DMSO) or HE-NECA in doses of 0.04 or 0.12 or 0.4 or 4 mg/kg were injected directly into the femoral vein in bolus of 2 mL/kg using an insulin syringe. Each mouse received one selected dose of HE-NECA or vehicle. Then BP was monitored at 5th, 15th, 30th, and 60th minute after the injection and three consequent BP measurements were performed for the each time-point. Data were computed as deltaBP (dBP) vs the basal BP measurement (Figure 6) as well as Mean Arterial Pressure (MAP) calculated as MAP = 2/3SBP + 1/3DBP (supplementary material Table S1).

### 5.12. Statistical Analysis

To test whether the data were normally distributed, the Shapiro–Wilk test was applied. Brown–Forsythe’s test was used to test for the homogeneity of variance, and Mauchley’s test for sphericity. Repeated measures one-way ANOVA with Sidak’s *post-hoc* test for multiple comparisons (only relevant comparisons were tested) applied in the in vitro aggregation experiments.

In the intravital microscopy experiments, the curves were fitted with a log(inhibitor) vs. response four-parametric equation with the use of GraphPad Prism v. 5 software. The Kruskal–Wallis test followed by Dunn’s multiple comparison test were used to compare the data regarding thrombus area.

Differences between the cumulative curves of the percentage of mice in which occlusion occurred with regard to time were determined by a time-to-event analysis and log-rank test; these comparisons were performed using Statistica v. 13.1 software. The significance of the differences between the ratios of We notice that you have uploaded supplementary material in the system which include a tale, however, we could not find citation of this supplementary table in your manuscript and we only find a citation of (sppl. mat. Figure) in Section 5.1. Please confirm the citation of supplementary table in main text and revise it accordingly the animals in which total occlusion occurred were tested with the use of Fisher’s exact *t*-test. Bootstrap approaches were employed to confirm whether the differences did not occur by pure chance and were not an effect of the small sample size.

The significance of differences between area or fibrinogen density of thrombi in the in vitro flow conditions were tested with Friedman’s test followed by Dunn’s multiple comparison test.

The statistical significance of the differences between the concentrations of fluorescent dyes used in blood–brain barrier permeability assessment was determined by two-way ANOVA followed by Tukey’s *post-hoc* test.

The significance of the hypotensive effect of HE-NECA compared to animals treated with a vehicle was evaluated with the Mann–Whitney U test.

## Data Availability

Data is contained within the article or Supplementary Materials.

## Supplementary Materials

The supplementary materials are available online at https://www.mdpi.com/1422-0067/22/6/3074/s1.
