# Supplementary material for: Adenosine Receptor Agonist HE-NECA Enhances Antithrombotic Activities of Cangrelor and Prasugrel in vivo by Decreasing of Fibrinogen Density in Thrombus
Source: Int J Mol Sci. 2021 Mar 17;22(6):3074. doi: 10.3390/ijms22063074 (PMC8002731; doi:10.3390/ijms22063074)
Supplement: Supplementary file 1 [file ijms-22-03074-s001.pdf]

Table S1. Maximal changes in Systolic (SBP) and Diastolic Blood Pressure (DBP) within 60 minutes after HE- NECA i.v. treatment shown as a delta (Δ) versus blood pressure value before the treatment.

	**VEH (DMSO 2ml/kg)**	**HE-NECA 4.0 mg/kg**	**HE-NECA 0.4 mg/kg**	**HE-NECA 0.12 mg/kg**	**HE-NECA 0.04 mg/kg**
**n =**	4	4	5	5	5
**Δ DBP±SD**	12.50±1.91	54.75±6.50*	40.40±9.73*	20.60±8.62	14.80±0.84
**Δ SBP±SD**	8.55±8.22	80.80±29.00**	30.90±8.65*	38.10±11.80*	17.30±6.36

Data shown as a Mean±SD; *p<0.05; **p<0.01 vs VEH; n=4-5.
